# Examining the efficacy of a cardio-dance intervention on brain health and the moderating role of ABCA7 in older African Americans: a protocol for a randomized controlled trial

**DOI:** 10.3389/fnagi.2023.1266423

**Published:** 2023-11-21

**Authors:** Mark A. Gluck, Joshua L. Gills, Bernadette A. Fausto, Steven K. Malin, Paul R. Duberstein, Kirk I. Erickson, Liangyuan Hu

**Affiliations:** ^1^Center for Molecular and Behavioral Neuroscience, Rutgers University-Newark, Newark, NJ, United States; ^2^Department of Kinesiology and Health, Rutgers University, New Brunswick, NJ, United States; ^3^Department of Health Behavior, Society and Policy, Rutgers School of Public Health, Piscataway, NJ, United States; ^4^AdventHealth Research Institute, Orlando, FL, United States; ^5^Department of Biostatistics and Epidemiology, School of Public Health, Rutgers School of Public Health, Piscataway, NJ, United States

**Keywords:** older adults, cognition, exercise intervention, older African American, clinical trial

## Abstract

**Introduction:**

African Americans are two to three times more likely to be diagnosed with Alzheimer’s disease (AD) compared to White Americans. Exercise is a lifestyle behavior associated with neuroprotection and decreased AD risk, although most African Americans, especially older adults, perform less than the recommended 150 min/week of moderate-to-vigorous intensity exercise. This article describes the protocol for a Phase III randomized controlled trial that will examine the effects of cardio-dance aerobic exercise on novel AD cognitive and neural markers of hippocampal-dependent function (Aims #1 and #2) and whether exercise-induced neuroprotective benefits may be modulated by an AD genetic risk factor, ABCA7 rs3764650 (Aim #3). We will also explore the effects of exercise on blood-based biomarkers for AD.

**Methods and analysis:**

This 6-month trial will include 280 African Americans (≥ 60 years), who will be randomly assigned to 3 days/week of either: (1) a moderate-to-vigorous cardio-dance fitness condition or (2) a low-intensity strength, flexibility, and balance condition for 60 min/session. Participants will complete health and behavioral surveys, neuropsychological testing, saliva and venipuncture, aerobic fitness, anthropometrics and resting-state structural and functional neuroimaging at study entry and 6 months.

**Discussion:**

Results from this investigation will inform future exercise trials and the development of prescribed interventions that aim to reduce the risk of AD in African Americans.

## Introduction

Older African Americans have two to three times higher rates of Alzheimer’s disease (AD) than White Americans (Lines et al., 2014). Lower socioeconomic status, as assessed by individual- (e.g., education, income) (Shadlen et al., 2006) and community-level variables (e.g., neighborhood-level indices of exposure to toxins and pollution, proximity to impoverished areas and crime) amplify this risk (Koster et al., 2005; Lines et al., 2014). Further, low levels of physical activity and exercise (Barnes and Bennett, 2014) due to, in part, adverse social influences on health [e.g., low levels of neighborhood walkability (Richardson et al., 2017; Rosso et al., 2021)] further accentuate AD risk (Jackson, 2003). However, epidemiological studies across non-Hipanic white, black, and Hispanic older adults demonstrate that individuals who engage in regular exercise in midlife have 37–66% lower odds of developing dementia (Ogino et al., 2019). Even among older adults who were previously sedentary, exercise begun later in life is associated with decreased AD risk (Ogino et al., 2019). Meta-analyses across exercise randomized controlled trials in adults show significant cognitive improvements in memory, attention, and executive function (Smith et al., 2010). As such, exercise is a promising strategy for attenuating cognitive decline and preventing AD, especially in older African Americans (National Academies of Sciences, Engineering, and Medicine, 2017).

Exercise is believed to improve cognition as well as memory through structural changes in the brain, namely increased hippocampus volume (Erickson et al., 2011; Maass et al., 2015; Makizako et al., 2015; ten Brinke et al., 2015). This is clinically relevant since the hippocampus—a brain region in the medial temporal lobe (MTL)–is especially vulnerable to damage in the earliest stages of AD. Interestingly, exercise may favor brain function prior to such structural changes (Stillman et al., 2016). For instance, aerobic exercise via walking or cycling-based interventions have shown benefit on functional connectivity (Hagan et al., 2016; Hsu et al., 2018; Prehn et al., 2019). In fact, a recent study on the effects of moderate-intensity cycle ergometry two times per weeks over 6 months reported increased resting state prefrontal cortex connectivity between key structures of executive function and default mode networks (Prehn et al., 2019). Another study reported that walking-based aerobic training for 3 days per week over 6 months among older adults with mild ischemia improved executive function and neural efficiency of associated brain areas (Hsu et al., 2018). However, prior studies focused on functional connectivity in the *prefrontal cortex*, not the hippocampus.

Moreover, few data exist on the effects of exercise on AD neuropathology in older adults, and no previous studies have examined neuropathological changes in African American samples or using the new blood-based biomarkers of AD. Although there is consistent evidence across several animal studies that exercise plays a role in modulating the AD neuropathological hallmarks, A*β* and tau (Adlard, 2005; Koo et al., 2017; Jeong and Kang, 2018; Brown et al., 2019), the conclusions from human studies are less clear. Baker and colleagues (Baker et al., 2010) detected a trend level decrease in plasma A*β* concomitant with significant executive function improvements among participants randomized to 6 months of aerobic exercise, relative to a stretching control group. More recently, Vidoni et al. (2021), reported no significant differences in amyloid PET change nor any cognitive changes among older adults randomized to a 52-week aerobic exercise program compared to an educational control group. Importantly, the field of AD research offers a broadly accessible, minimally invasive, inexpensive method to screen cerebral A*β* and tau pathologies: blood-based biomarkers for AD. To date, however, underrepresented populations have rarely been studied using culturally sensitive exercise programs with these new types of blood-based biomarker outcomes (Colcombe et al., 2004; Voss et al., 2010).

Tailoring exercise to underrepresented groups may be important for adherence purposes as well as for improved cognition. Recently, an 8-month Latin dance program improved working memory among middle-aged and older Latinos (Aguiñaga et al., 2022). This is in line with another study reporting greater composite executive function and digit symbol substitution scores in the ballroom dancing treatment compared to a treadmill group in at-risk multi-race population for dementia (Blumen et al., 2022). Furthermore, these cognitive effects corresponded with less hippocampal atrophy in the ballroom dancing compared to the treadmill group (Blumen et al., 2022).

A previous study from our laboratory at Rutgers University-Newark’s *Aging and Brain Health Alliance* recruited older African American participants and demonstrated that 2 days/week of cardio-dance fitness (CDF) classes over 5 months resulted in improved generalization. Generalization is the ability to apply previously learned rules to new situations and contexts (Myers et al., 2002, 2008; Sinha et al., 2021), a cognitive function supported by the MTL including the hippocampus (Myers et al., 2008). Further, we found that CDF increased the dynamic rearrangement (network flexibility) of resting-state networks within the MTL (Sinha et al., 2021). Given previous findings suggesting generalization is impaired in preclinical AD (Myers et al., 2008), these later results suggest MTL dynamic network flexibility may be a novel mechanism through which exercise improves cognition and reduces AD risk. Not all individuals gain neuroprotective benefits from aerobic exercise, however. ABCA7, or adenosine triphosphate (ATP)-binding cassette member, is a risk gene associated with late-onset AD (Tosto and Reitz, 2013). Our previous findings suggest older African Americans with the risk variant of the ABCA7 (single-nucleotide polymorphism rs3764650) gene derive less cognitive benefit from exercise (Sinha et al., 2020). However, our previous study had several limitations including: limited sample size to elucidate the effect of ABCA7 on exercise-induced changes in MTL dynamic network flexibility; no active control group; and lack of correspondence with the public health recommended guidelines of 150 min of moderate-to-vigorous activity per week. These limitations hindered the ability to detect the impact of recommended amounts of aerobic exercise on brain health. However, our twice a week CDF classes were well received with positive feedback and low dropout rates. Participants indicated they would attend an additional class if provided in a post-intervention survey.

In summary, it remains an open question whether the cognitive benefits of exercise—and accompanying neural changes—are: (1) compensatory in nature (and, thus, do not affect underlying AD neuropathology), or (2) disease modifying such that exercise attenuates the progression of or possibly reverses underlying AD neuropathology. Thus, we describe the protocol for a Phase III randomized controlled trial (NCT05597124; 1R01AG078211) that will examine the effects of a CDF intervention versus an active comparator arm using strength, flexibility, and balance on novel AD cognitive and neural markers of hippocampal-dependent function. Specifically, we will examine generalization of prior learning and MTL dynamic network flexibility (Aims #1 and #2, respectively) and whether exercise-induced neuroprotective benefits may be modulated by ABCA7 rs3764650. We will also explore the effects of CDF on blood-based AD neuropathology biomarkers (i.e., amyloid pathology, tau pathology, neurodegeneration and neuroaxonal injury).

## Methods and analysis

### Participants

This randomized controlled trial (RCT) intends to enroll 280 older African Americans into a 6-month intervention. Participants will be recruited in the Greater Newark, NJ area by the Aging and Brain Health Alliance at Rutgers University-Newark, a university-community partnership fostered over 17 years of ongoing community engagement, health education, and service. Community partners include leadership and members of public and subsidized housing, local churches, mosques, senior centers, and health and wellness organizations that serve Greater Newark, NJ. Recruitment methods are described elsewhere (Gluck et al., 2019; Esiaka et al., 2022).

The eligibility criteria were designed to balance safety and generalizability (Table 1). All potential participants will be screened via telephone to determine whether they meet inclusion criteria.

**Table 1:** Inclusion and exclusion criteria.

Inclusion	Exclusion
Self-identify as either African American or Black ≥60 years of age Able to speak, read, and understand English Available over the study period (6 months) Independently ambulatory (i.e., not needing a wheelchair, walker, or cane) Meet criteria for low levels of physical activity (<60 min/week) based on the International Physical Activity Questionnaire (IPAQ-short version) Scoring 28–35 (inclusive) on the Telephone Interview for Cognitive Status Modified Education adjusted Montreal Cognitive Assessment score of 20–26 (inclusive) Have clearance to participate from their primary care physician	Color-blindness (because some of the cognitive tasks utilize color as a cue) Any diagnosed neurological disorder (including headaches and peripheral neuropathy); Diagnosed or self- reported non-neurological conditions that likely affect MTL outcomes, such as, major depressive disorder or a Geriatric Depression Scale-Short Form score ≥ 5 Schizophrenia, delusional disorder, schizoaffective disorder or significant psychiatric symptoms that could impair the completion of the study (e.g., psychosis) Substance-related and addictive disorders (or treatment in past 5 years) Active chemotherapy or radiation treatment for cancers (within the past 3 months) Planning to undergo general anesthesia during the next 6 months. Exercise contraindications, such as, orthopedic complications, myocardial infarction, coronary artery bypass grafting, angioplasty or other cardiac condition in the past year, current treatment for congestive heart failure, angina, uncontrolled arrhythmia, deep vein thrombosis (DVT) or another cardiovascular event, and uncontrolled hypertension with resting systolic or diastolic blood pressures >180/110 mmHg Presence of metal implants (pacemaker, stents) or metal on the body that are MRI contraindicative Claustrophobia Decline participation in blood work/draw procedures. Are underweight and are unable to participate in blood work/draw procedures Have diabetes and are receiving insulin, sulfonylureas, or have poor uncontrolled Hemoglobin A1c levels (>8.5%). Unwilling to be randomized to either intervention group Resides outside the Greater Newark area Self-reported fall history—more than once in the past 6 months Planned travel for 7+ consecutive days of travel over the next 6 months

### Procedure

**Figure 1:**
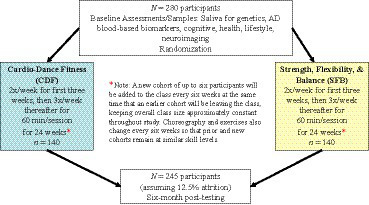
Study design.

After passing the initial telephone screening, participants will be invited to complete a study entry assessment involving blood and saliva collection; health, lifestyle, and socio-demographic questionnaires; cognitive testing; physical activity and physical function measures; and a brain imaging session (Figure 1). All assessments will be administered by certified testers who have undergone rigorous training to ensure consistency of test administration across trial (Table 2). To further achieve internal validity and inter-rater reliability, the testers will observe and evaluate each other at least once per quarter and audit each other’s testing records.

After the study entry assessments, enrolled participants will be randomly assigned to (1) Cardio-Dance Fitness (CDF), an aerobic exercise program of initially 2 then 3 days/week for 60-min/session, resulting in 150 min/week of aerobic activity (*n* = 140); or (2) Strength, Flexibility & Balance (SFB) low-intensity movement of initially 2 then 3 day/week for 60-min/session (*n* = 140). A stratified permuted block randomization algorithm with equal (1:1) allocation will be used to assign participants to the CDF or SFB intervention. To maintain allocation concealment, random block sizes will be used. Randomization will be stratified by age (60–75 or > 75 years) and sex. Unblinded staff will implement the randomization. Participants will be informed of their assigned group after completion of all study entry assessments. To achieve unbiased results, the PI, as well as the testers/outcome assessors, will be blinded to group assignment.

**Table 2:** Summary of measures collected at each study visit.

Category and measurement	Study entry	24-weeks
Informed consent	Yes	No
Inclusion, sociodemographic, psychosocial and other measures		
Personal/demographic data	Yes	No
Health and Lifestyle Questionnaire	Yes	Yes
Geriatric Depression Scale-Short Form	Yes	Yes
Midlife Development Inventory	Yes	Yes
Perceived Stress Scale	Yes	Yes
Morningness-Eveningness Questionnaire	Yes	Yes
Social Network Index	Yes	Yes
Exercise Social Provisions Scale	Yes	Yes
Ishihara’s Tests for Color Deficiency	Yes	No
Telephone Interview for Cognitive Status-Modified	Yes	No
Montreal Cognitive Assessment	Yes	No
International Physical Activity Questionnaire	Yes	No
Cognitive measures		
Rey Auditory Verbal Learning Test	Yes	Yes
WAIS-IV Digit Span^a^	Yes	Yes
Trail Making Test	Yes	Yes
Controlled Oral Word Association	Yes	Yes
Rutgers Generalization Tasks	Yes	Yes
Physical function and biometric measurements		
Six-Minute Walk Test	Yes	Yes
Blood pressure	Yes	Yes
Heart Rate	Yes	Yes
Body mass index	Yes	Yes
Waist-to-hip ratio	Yes	Yes
Brain imaging protocol		
fMRI (ADNI 4 protocol + High-Resolution Analyses)^b^^,^^c^	Yes	Yes
Structural imaging	Yes	Yes
Blood and saliva collection		
Genetics (ABCA7-50) via saliva	Yes	No
P-tau217^d^	Yes	Yes
P-tau231	Yes	Yes
Neurofilament light	Yes	Yes
AB 42/40 ratio^e^	Yes	Yes

a Weschler Adult Intelligence Scale Fourth Edition.

b Functional Magnetic Resonance Imaging.

c Alzheimer’s Disease Neuroimaging Initiative.

d Phosphorylated Tau.

e Beta Amyloid plaque.

### Inclusion, sociodemographic, psychosocial, and other measures

#### Personal/demographic data

Age, years of education, primary occupation, current work status, marital status, median household income.

#### Health and lifestyle questionnaire (5 min)

A brief questionnaire will be completed for descriptive purposes to identify health and lifestyle factors such as diet, alcohol use, sleep, smoking habits, weekly exercise and activity levels (Washburn et al., 1993).

#### Midlife development inventory (MIDI) (15 min)

The MIDI is a personality scale that captures the ‘Big 5’ personality traits: Neuroticism, Extraversion, Openness to Experience, Agreeableness, and Conscientiousness. Participants rate on a scale of 1–4 (“a lot” to “not at all”) how well each of the 31 adjectives describes them (Lachman and Weaver, 1997).

#### Perceived stress scale (5 min)

Participants will respond to 10 items on a 5-point Likert scale (0-never to 4-very often) regarding their feelings and thoughts during different situations in the past month. Total scores range from 0 to 40 with higher scores indicating higher perceived stress levels (Cohen et al., 1983).

#### Geriatric depression scale-short form (5 min)

A measure of depressive symptomology, participants respond yes or no to 15 items related how s/he felt during the past week. Total scores range from 0 to 15. Participants who score ≥5 points are deemed ineligible and referred to their primary care physician or the study physician (if they do not have a primary doctor) for follow-up (Yesavage and Sheikh, 1986).

#### Social network index (10 min)

Cohen’s Social Network Index will be administered to measure both the size and composition of a participant’s social network. Social network size will be summed as the number of living children, relatives other than children, and friends with whom the participant feels close. Higher numbers indicate a larger social network. Social network composition will be the number of social roles in which the participant has regular contact (i.e., once every 2 weeks) across 12 categories of social relationships: spouse, parent, child, child-in-law, close relative, close friend, church/temple member, student, employee, neighbor, volunteer and group member. Higher numbers reflect greater diversity in one’s social network. Both social network size and composition will be used for descriptive purposes as well as potential covariates (Cohen et al., 1997).

#### Exercise social provisions scale (10 min)

Participants will respond to 24 items related to their social relationships within the exercise program (e.g., I feel part of a group of people who share my attitudes and beliefs) at study entry and at 6-month follow-up. This measure will be used for descriptive purposes (Jones and Perlman, 1987).

#### Ishihara’s tests for color deficiency (2 min)

Given that our cognitive tasks involve color discrimination, normal or near-normal color vision is a crucial inclusion criterion. This test will identify color perception and red-green color deficiencies. This is an inclusion measure (Hardy et al., 1945).

#### Telephone interview for cognitive status-modified (10–15 min)

The Telephone Interview for Cognitive Status-Modified is a phone-based assessment of cognitive function. Cutoff scores of 28–35 (inclusive) will be used to screen individuals with probable mild memory deficits (Knopman et al., 2010).

#### Montreal cognitive assessment (5–10 min)

This test will assess participants’ global cognitive status. Cutoff scores of 20–26 (inclusive) will be used to identify individuals with probable mild memory deficits (Nasreddine et al., 2005).

#### International physical activity questionnaire-short form (5 min)

This questionnaire will assess participants’ subjective report of the amount, frequency, and intensity of different types of physical activity (moderate, vigorous, walking) as well as sitting time engaged in over the past 7 days. Used as a screening measure, individuals will be eligible if they meet criteria for low levels of physical activity (<60 min/week) (Lee et al., 2011).

### Cognitive measures

#### Rey auditory verbal learning test (RAVLT) (10–15 min)

This test starts with 15 words read aloud by the examiner at the rate of one per second. The participant repeats all the words he/she can remember, in any order. This is repeated five times. Then, the examiner presents a second list of 15 new words, allowing the participant only one attempt at recall. Following this, the participant is asked to recall as many words as possible from the first list. After a 20-min delay, the participant is again asked to recall the first list. The RAVLT measures short-term auditory-verbal learning and memory, rate of learning, proactive and retroactive interference, confabulation in memory, retention of information, and differences between learning and retrieval (Schmidt, 1996).

#### WAIS-IV digit span (10–15 min)

Participants will read lists of numbers and then asked to repeat them back in the same order, the reverse order, or numerical order. This assesses rote learning, memory, attention, auditory processing, mental manipulation, and working memory (Kumar and Priyadarshi, 2013).

#### Trail making test A and B (3–5 min)

This is a neuropsychological test of visual attention and task switching. It consists of two parts in which the subject is instructed to connect a set of 25 dots as quickly as possible while still maintaining accuracy. The test can provide information about visual search speed, scanning, speed of processing, mental flexibility, as well as executive functioning. It is sensitive to detecting cognitive impairment associated with dementia and AD (Arnett and Labovitz, 1995).

#### Controlled oral word association (5 min)

This test consists of three-word conditions. The subjects’ task is to produce as many words as they can that begin with the given letter (F, A, or S) within a 1-min time period. Subjects are also instructed to exclude proper nouns, numbers, and the same word with a different suffix. It is a commonly used neuropsychological measure of verbal fluency and has been shown to be a sensitive indicator of frontal lobe dysfunction (Golden et al., 2000).

#### Rutgers generalization tasks (30 min)

We will administer two *Rutgers Generalization Tasks*, cognitive assessments developed by the PI (Gluck) and colleagues, derived from predictions of their neurocomputational models of the entorhinal cortex and hippocampus in stimulus representation. These are measures of generalization which will be used to test the effect of the CDF intervention on cognition at study entry and 6 months (Myers et al., 2002, 2003, 2008).

##### Rutgers generalization task #1: acquired equivalence task

**Figure 2:**
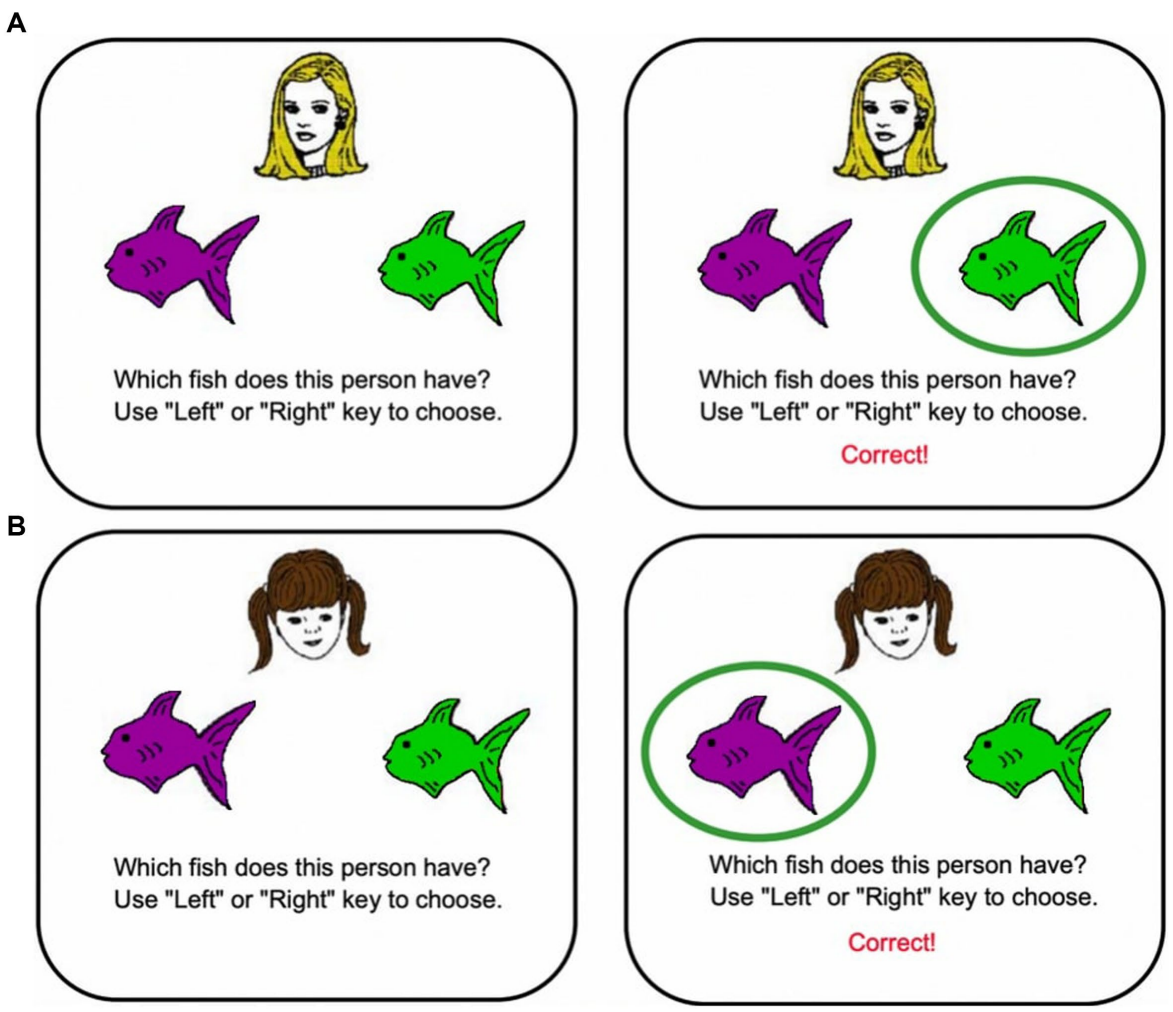
Acquired equivalence task.

Methods are previously described elsewhere (Myers et al., 2003). In brief, participants first learn to form associations between various faces and fish via trial-and-error learning. On each trial, participants see a cartoon face and two different colored fish, and guess which fish belongs with each face (Figure 2). During learning, participants learn to pair Face A with Fish 1, then to pair Face B also with Fish 1. In the process, participants should learn that Face A and Face B are equivalent with respect to what color of fish they are paired with. Next, participants learn to pair Face A with a new fish, Fish 2. During a final generalization stage, feedback is not provided. Participants are tested on transfer of their knowledge to novel pairings; if they previously learned that Face A and Face B were equivalent, then participants should generalize that Face B is paired with Fish 2, even though they have not yet been presented with this particular pairing. Gluck’s theoretical models (Moustafa et al., 2010), as well as animal lesion studies using a homologous rodent task (Coutureau et al., 2002), suggest that Acquired Equivalence depends on the entorhinal cortex (EC) and parahippocampal regions of the MTL. Individuals with structural changes in the MTL associated with prodromal AD show normal learning but are selectively impaired on generalization (Myers et al., 2002, 2003; Bódi et al., 2009), while generalization is unimpaired in healthy aging (Raz, 1997).

##### Rutgers generalization task #2: concurrent discrimination and transfer task

**Figure 3:**
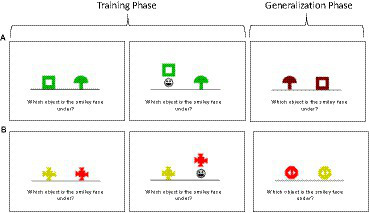
Concurrent discrimination and transfer task.

Methods are as previously described (Myers et al., 2002). In brief, on each trial, subjects see a pair of colored shapes and learn to choose the correct objects from each pair via feedback; the chosen object is raised and, if the response is correct, a smiley face is revealed underneath (Figure 3). Within each pair, the objects differ in color or shape but not both. For each discrimination pair, one feature, color or shape, is relevant and one irrelevant. As shown in the row A, one pair might involve learning to choose a green square over a green mushroom. In this example, shape (square vs. mushroom) is relevant, but color is irrelevant for predicting the correct answer. This training phase is followed by a generalization phase, in which the irrelevant features are changed but the relevant features remain the same. Thus, the discrimination rules have not changed from learning to transfer, only the irrelevant features changed. Individuals who learned based on relevant features only should continue to perform well in the generalization phase. In contrast, individuals who learned based on all the features equally, are effectively confronted with novel objects in the transfer phase and should perform near chance. Performance on the transfer phase distinguishes between hippocampal-atrophied and non-atrophied individuals (Myers et al., 2002, 2008).

### Physical activity and physical function

#### Six-minute walk test (6MWT) (10–15 min)

Participants are instructed to walk a pre-measured length on a flat surface (e.g., a 30-meter loop) for 6 min, covering as much distance as possible. To be collected for descriptive purposes, the 6MWT approximates participants’ maximal oxygen consumption as a measure of physical fitness and has excellent test–retest reliability (ICC = 0.95) and convergent validity with other measures of physical functioning (Borg, 1982).

#### Physical measurements (5–10 min)

For descriptive purposes, we will measure blood pressure, heart rate, body mass index, and waist-to-hip ratio at study entry and six-month post-testing (post-testing at least 24–72 h after the last bout of training to avoid residual effects).

##### Blood pressure (BP)

BP will be assessed in a sitting position after a 5-min resting period. Blood pressure will be assessed using the automatic Mircolife Blood Pressure Monitor (Microlife USA, Inc., Clearwater, FL). During each visit, three consecutive BP readings were obtained on the right arm in a seated position. BP will be calculated as the mean of the lowest two BP readings which is aligned with the American Heart Association’s Guidelines if they are within 5 mmHg of each other to ensure accuracy.

##### Heart rate (HR)

HR will be assessed using the Mircolife Blood Pressure Monitor (Microlife USA, Inc., Clearwater, FL) after a 5-min resting period. HR will be assessed twice and averaged if measure does not exceed 5 beats per minute (bpm). If not, a third HR will be assessed and averaged with the closest HR measure (<5 bpm).

##### Body mass index (BMI)

Weight and Height will be assessed using the SECA 777 Digital Scale (SECA., Hamburg, Germany). Weight will be measured to the nearest 0.1 kg. Height will be measured to the nearest 0.1 cm. BMI will be calculated as the participants weight in kilograms divided by the square of height in meters. Height and weight will be assessed twice and averaged if height does not exceed 0.5 cm and weight does not exceed 0.1 kg. If not, a third measure of both will be assessed and averaged with the closest height and weight measures (<0.5 cm and 0.1 kg).

##### Waist-to-hip ratio (WHR)

Waist circumference will be measured with the participant standing upright, with their arms at the sides of the body and feet together. A horizontal measurement will be taken at the narrowest part between the umbilicus and xiphoid process of the torso. Hip circumference will be assessed with the participant standing with their legs slightly a part. A horizontal measurement will be taken at the maximal circumference of the hip. Then, waist-to-hip ratio (WHR) will be calculated as a ratio measurement of the circumferences of the waist to that of the hip by the following equation: WHR = waist circumference / hip circumference (American College of Sports Medicine, 2018). Waist and hip circumference measurements will be assessed twice and averaged, both measures must be within 0.5 cm of each other. If not, a third measure will be assessed for both sites and it will be averaged to the closest measurement (<0.5 cm).

### Brain imaging protocol

#### ADNI 4 protocol + high-resolution analyses for fMRI

Participants will undergo whole- brain structural and resting-state fMRI. *MRI Acquisition Methods*: All MRI scans will be performed on a Siemens 3T Magnetom PRISMA equipped with state-of-the-art 32-channel Multiband parallel encoding head coils. We use the ADNI 4 protocol to match the state of the art in the field of AD research. High-resolution resting-state functional scans will also be acquired for each participant.

#### Structural MRI analysis

We will use Advanced Normalization Tools (ANTS) (Avants et al., 2011), which implements a powerful diffeomorphic registration algorithm known as Symmetric Normalization (SyN) (Klein et al., 2009). The Diffeomorphic Registration based Cortical Thickness (DiReCT) technique uses SyN to warp each scan’s gray/white matter boundary to the gray/CSF boundary, and uses the inter-boundary distance to estimate thickness within specified anatomical constraints (Das et al., 2009). The latter prevents the algorithm from over-estimating thickness in regions with deep sulci or intersecting gray matter banks and corrects for segmentation errors. Our focus will be on measuring cortical thickness of the entorhinal cortex (EC), especially the anterolateral EC. Other analyses will also include hippocampal subfield morphometry and volumetry (Yushkevich et al., 2010).

#### Functional MRI analysis

Scans will undergo motion correction, structural co-registration, and cross-participant diffeomorphic registration using ANTS. The effects of head motion, mean global signal, ventricular mean signal, and white matter mean signal will be regressed out. MTL sub-regions will be segmented based on published protocols and the mean time series will be extracted for each region of interest (ROI) (Reagh et al., 2018). ROIs include: hippocampal subfields (subiculum, CA1, and DG/CA3), and cortical regions (perirhinal cortex, parahippocampal cortex, anterolateral entorhinal cortex, and posteromedial entorhinal cortex). To assess dynamic connectivity between the ROIs, we will use a Louvain-like locally greedy community detection algorithm for optimizing multilayer modularity which quantifies network community structure and reconfiguration over time (Mucha et al., 2010; Braun et al., 2016). Optimization of multilayer modularity will yield a community assignment for every node and time window, indicating module allegiance. To measure changes in node communities across time, we will compute flexibility of each node as the extent to which it changed module allegiance throughout the set of time windows represented by the multilayer network (Bassett et al., 2011). Flexibility is quantified as the number of times a node changes community assignment, normalized by the total possible number of changes, for each MTL ROI. The flexibility of the MTL network will then be computed as the mean flexibility over all nodes.

To ensure safety in the MRI scanner, all potential participants will be screened to determine whether they can safely enter the MRI environment. Participants that are claustrophobic or have any magnetic metal such as iron, nickel or cobalt implanted in or on their body, hair, or apparel including metal flakes or filings, surgical pins or plates, electrical devices such as a pacemaker, jewelry, or metal ink facial/ neck tattoos will be excluded from this study.

All participants who elect to participate in MRI testing will be provided with a detailed explanation of the procedure and will be informed that they may choose to discontinue the procedure at any time.

### Blood and saliva collection

Blood samples will be collected by a phlebotomist at the Clinical Research Unit of the Rutgers New Jersey Medical School (NJMS). One 10 mL tube of EDTA blood will be collected and labeled with the participant ID number and date of collection in front of the participant as per procedures by the Clinical Research Unit of the Rutgers NJMS. Blood samples will be centrifuged within the hour of blood draw. Two aliquots of EDTA plasma collected will be transferred to a 1.5 mL Olympus microtube, and frozen at −80°C. The frozen plasma will be shipped via World Courier^1^ on dry ice overnight in a well-sealed/taped Styrofoam box with a tracking number. The EDTA plasma will be sent to Clinical Neurochemistry Lab at University of Gothenburg for processing and assaying for the following AD neuropathology markers using ultrasensitive Single molecule array technology: P-tau217, P-tau231, and AB 42/40 ratio. Plasma neurofilament-light chain (NfL) levels will also be assessed. Our University of Gothenburg collaborators’ unique expertise in blood-based biomarkers for AD ensures the highest quality analyses, while their input into the interpretation of results will be key. Moreover, their involvement ensures that our project remains at the innovative leading edge should newer AD neuropathology biomarkers emerge.

Saliva samples will also be collected at CRU using Oragene-DNA (OG-600) self-collection saliva kits. Immediately after collection, the saliva samples are temporarily stored at room temperature in the Clinical Research Unit of the Rutgers NJMS. Once a week, Biosafety Transportation (IATA/ICAO) certified key personnel will bring the saliva samples back to the laboratory where they will be stored at room temperature. Once a quarter, Biosafety Transportation (IATA/ICAO) certified key personnel will ship the collected saliva samples to Sampled SMART Labs in Piscataway, NJ for processing and AD risk genotyping (ABCA7, APOE-ε4).

### Measures assessed during interventions

The following measures will be administered during the exercise intervention among randomized participants.

### Heart rate monitoring

At each exercise class, participants will wear a Fitbit Versa 2 HR sensor on their non-dominant wrist to measure HR. To assure scientific rigor, the Administrative Assistant will record average HR to ensure that moderate-intensity (50–75% HR max) aerobic thresholds are achieved for the aerobic CDF condition and below aerobic thresholds (50% HR max) are maintained for the low intensity SFB condition. HR max is measured 220 – Age based on the CDC criteria. HR will be measured at: (1) the beginning of a session, (2) at the 25-min mark during the 50 min of aerobic CDF exercise (or the equivalent 25-min mark for the SFB group), (3) 2-min post the full 50 min of aerobic CDF exercise (or equivalent 2-min post for the SFB group), and (4) at the end of class.

### Borg ratings of perceived exertion (RPE)

Participants will report their RPE, which is their perception (on a 15-grade scale) of how much effort they are exerting during the exercise sessions at (1) the beginning of a session, (2) at the 25-min mark during the 50 min of aerobic CDF exercise (or the equivalent 25-min mark for the SFB group), (3) 2-min post the full 50 min of aerobic CDF exercise (or equivalent 2-min post for the SFB group), and (4) at the end of class.

### Data and safety monitoring plan

The investigators will monitor participant safety on a case-by-case basis and discuss the overall safety of the study on a weekly basis. The PI and Co-Investigators will also teleconference with the DSMB and NIA officials every 6 months to provide an added layer for monitoring safety and adverse events. The PI will be notified (by study coordinators) of any Serious Adverse Events as soon as they occur and will notify the DSMB, IRB and NIA within 24 h of learning about the event.

### Adverse event (AE) reporting

Participant-facing staff [i.e., testers, interventionists (Fitness Instructors, exercise site research assistant), community brain health educator] will watch the NIA Safety Training Course video and be trained to collect and report AEs using a standard form adapted from the NIH Clinical Research Toolbox. We will use the NIA standard definitions of AEs from the “2018 NIA Adverse Event and Serious Adverse Event Guidelines.” Any adverse events will be reported to the Institutional Review Board and the DSMB within 48 h of learning of the event by the PI. In the unlikely event of a serious adverse event, the PI will report the event to the IRB, NIH Project Officer, and DSMB within 24 h of learning of the event.

### Intervention procedures

#### Cardio-dance fitness (CDF)

This is an aerobic CDF exercise class in a social context with aerobic intensity assessed by heart rate monitoring throughout the class. Participants will meet for approximately 60 min/session, over 24 weeks (6 months). As advised by the American College of Sports Medicine (ACSM) for sedentary older adults, both intensity and duration will be gradually ramped up for 3 weeks. The classes will be offered, initially, 2 day/week and then shift to 3 day/week in the fourth week. Study coordinators and administrators will ensure participants show up for the appropriate number of classes. Participants who complete ≥80% of the classes (i.e., at least 12) will be considered fully adherent to the protocol (55 of 69 total classes). Participants will begin by reaching 50–60% of age-adjusted predicted heart rate max for 20–30 min/session, and then incrementally increasing intensity and duration over the first few weeks toward 75% intensity, as well as a Rating of Perceived Exertion (RPE) of 13–14, for 50 min/session. Once at 75% intensity, participants will do 5 min of warm-up, 50 min of aerobic (CDF) exercise and 5 min of cool down, all led by a Certified Fitness Instructor blinded to study hypotheses and following ACSM guidelines. An assistant assigned to each class will take attendance and record both heart rates and RPE. A new cohort (of 4 to 5 people with a target female to male sex ratio of 2:1) will be added to the class every 6 weeks while an earlier cohort will be leaving the class, keeping the overall class size approximately constant throughout most of the study. An additional staff member will attend every class to closely monitor heart rate and RPE of these new participants, all of whom will be physically separated from the rest of the class in the back. The assistant will instruct these new participants to relax or cease movement for a few minutes, throughout the initial weeks of ramping up. This procedure ensures that the prior cohort(s) are exposed to a full 50-min session of aerobic exercise, while the new cohort receives more attention from study staff resulting in shorter and less intense ramp-up sessions for them. In addition, the instructor will change the choreography every 6 weeks to ensure that participants in prior cohorts are at a similar skill level as the new cohort on the current choreography. Ultimately, cohorts rotate out of the classes after completing 24 weeks of CDF. Participants will have additional incentives (compensation and gifts) for attending 80% of classes, parking will be provided at the site of class, water will be available on site, participants with diabetes will be provided snacks upon request.

#### Strength, flexibility and balance (SFB)

This intervention will serve as a rigorous, structurally equivalent, active comparator to the CDF intervention, identical in duration, frequency, and social contact except that the group will engage in non-aerobic activities. Structural equivalence ensures that our inferences about the CDF group can be attributed to the aerobic component rather than duration, frequency, or within- class social contact. Participants who complete ≥80% of the classes (i.e., at least 12) will be considered fully adherent to the protocol. SFB will involve non-aerobic activity with strength, flexibility, and balance training. Equipment which will be utilized consists of light dumbbells (3-5lbs), resistance bands, and yoga mats. The assistant will ensure participants remain below aerobic thresholds, namely less than 60% of target heart rate max and a RPE between 9 and 10, during all sessions. The sessions will include 5 min of warm-up, 50 min of non-aerobic exercise, and 5 min of cool down. As with CDF, new cohorts will begin every 6 weeks. Participants will have additional incentives (compensation and gifts) for attending 80% of classes, parking will be provided at the site of class, water will be available on site, participants with diabetes will be provided snacks upon request.

### Overview of study aims and predictions

The aims of this study are to:

Aim #1: Examine the effect of an exercise dance intervention on changes in a cognitive marker of AD, generalization (timeframe: baseline and 6 months). *Prediction 1*: We expect that participants assigned to the CDF group will show greater improvements in performance on generalization compared to SFB group after 6 months (*primary outcome*).
Aim #2: Examine the effects of an exercise dance intervention on changes in a fMRI biomarker of AD, MTL network flexibility (timeframe: baseline and 6 months), and determine whether improvements in network flexibility mediate CDF-improvements in generalization. *Prediction*: We expect that the CDF group will show greater improvements than the SFB group in MTL network flexibility (*co-primary outcome; Prediction 2.1*), which, in turn, will statistically mediate improvements in generalization after 6 months (*Prediction 2.2*).
Aim #3: Examine whether the ABCA7 genotypic variations modulate the influence of CDF intervention on cognitive (Aim #1 outcome) and neural (Aim #2 outcome) markers of AD risk (timeframe: baseline and 6 months). *Prediction 3*: We expect a *genotype x intervention group x time* interaction such that post-exercise improvements in generalization and network flexibility will be observed selectively in carriers of the ABCA7 non-risk genotypes after 6 months.
Exploratory Aim #4: Explore the effects of the CDF intervention on blood-based AD biomarkers (plasma p-tau217, 231), and how these relate to changes in generalization and network flexibility (timeframe: baseline and 6 months). Plasma neurofilament light chain changes will also be explored and related to changes in generalization and network flexibility. *Exploratory Analyses*: This aim will be used to further understand if the aerobic intervention modifies AD neuropathology or whether the cognitive and/or neural benefits are compensatory in nature (contribute to cognitive reserve but do not change underlying AD neuropathology).

### Data analysis

We will perform generalized mixed models, based on the various independent/covariate measures and on our primary outcome variables. Our primary (behavioral) outcome variable is generalization (hippocampus-related cognitive function), continuous measure, as measured by the Rutgers Generalization Tasks: (1) Acquired Equivalence Tash (2) Concurrent Discrimination and Transfer Tasl. Our co-primary (neural) outcome variable is MTL network flexibility, continuous measure, from resting-state fMRI. We expect MTL network flexibility to act as a mediator, and ABCA7 genotype to act as a moderator, for CDF-related improvements in generalization.

#### Statistical data analysis plan

For each Aim, summary statistics and histograms will be calculated and plotted to explore the differences in the distributions of data, cross-sectionally and longitudinally, between intervention conditions (CDF vs. SFB). Although we do not anticipate study entry differences in the distribution of measured factors between treatment conditions, we will assess statistical differences using appropriate parametric (e.g., t test) and non-parametric tests (e.g., Mann–Whitney *U* test). Variables that show a study entry difference (*p* < 0.10) will be controlled as potential confounders in statistical analysis described below, when appropriate. Alternatively, we will construct propensity scores and control for the propensity scores as a covariate in the statistical models to control for any observed imbalances in study entry variables (Leyrat et al., 2013). For each test of an outcome, we define the statistical significance by *p* < 0.05. False discovery rate will be applied for multiple testing as appropriate (Benjamini and Hochberg, 1995).

An intent-to-treat approach will be used in all primary analyses. Per protocol analyses will further examine effects of CDF relative to SFB among those who are adherent (i.e., who completed 80% or more classes) in both conditions. To optimally handle missing data and maximize the utilization of information, we will employ statistical techniques such as maximum likelihood estimation methods, which efficiently incorporate partially observed data. We will use linear mixed models for data analysis, providing flexibility in specifying the variance/covariance structure for the random effects and errors. This approach often leads to improved fit of the analytical model to the actual data.

For Aim 1, we will use mixed model analysis to account for the repeated measures design, with generalization (continuous variable) as the dependent variable, and include group (CDF vs. SFB), time, and group × time interaction as fixed effect independent variables. Participants will be treated as random effects. Age, sex, and study entry variables that show a difference of *p* < 0.1 (or propensity score) will be controlled as covariates in the statistical model. For Prediction 1, we will construct linear contrasts to compare the changes in generalization from study entry to 6 months between CDF and SFB.

For Aim 2 Prediction 2.1, a mixed model analysis will compare MTL flexibility (co-primary outcome) between CDF vs. SFB. For Aim 2 Prediction 2.2, we will establish the mediation model to study if changes in generalization following the CDF exercise intervention will be mediated by increases in fMRI biomarker levels of network flexibility. We outline the conceptual framework of mediation analysis using linear regression, while the analysis will be performed using mixed model analysis (Golden et al., 2000), as follows: let “O” denote the outcome (e.g., generalization), “T” denote the independent variable (treatment) and “M” the mediators (network flexibility). The first model is an association model of T and M, i.e., M = γ_0_ + γ_1_T + ε* and the second model is the mediational model: O = β_0_ + β_1_T + β_2_M + ε, where ε and ε* are random errors independent of T and M. The mediation effect will be evaluated by testing H_0_: γ_1_β_2_ = 0, using the bootstrap method (Myers et al., 2003) to construct the 95% confidence interval (CI). If the 95% CI does not include 0, we will reject H_0_ and establish the mediation model. Moreover, we will examine β_1_ to evaluate how it changes with M vs. without M, in terms of its magnitude, direction and value of *p*, and calculate effect size of mediation as P_M_ = γ_1_β_2_/α_1_, where α_1_ is the regression coefficient in O = α_0_ + α_1_T + ε (Moustafa et al., 2010).

For Aim 3 Prediction 3, we will conduct two separate mixed model analyses, one with generalization as the dependent variable, and the other with network flexibility. In each model, we will include genotype (ABCA7 non-risk vs. high-risk), group (CDF vs. SFB), time (study entry vs. 6 months), and all the two-way and three-way interactions of genotype, group, and time interactions as fixed effect independent variables. Variables such as age, gender, and study entry variables that show a difference of *p* < 0.1 (or propensity score) will be controlled as covariates in the model. The interaction of genotype × group × time will be tested using Type 3F test.

For Exploratory Aim 4, following a similar mixed model analysis as in Aim 1, we will construct linear contrasts to estimate and compare the changes in plasma Aβ 42/40, p-tau217, p-tau231, and neurofilament light (secondary outcomes) between CDF vs. SFB. Changes in blood biomarkers will be correlated with behavioral generalization and MTL flexibility change scores; age, sex, and study entry variables that show a difference of *p* < 0.1 (or propensity score) will be controlled as covariates in the correlation model.

##### Power and sample size considerations

We plan to enroll 280 individuals with 140 individuals per randomized group (CDF and SFB). In the following, we estimated the minimal effect sizes (Cohen’s d and f^2^) with *n* of 244 participants (122 per group) before accounting for ~12.5% attrition rate. For Aim #1 Hypothesis 1 and Aim #2 Hypothesis 2.1, with *n* = 122 per group, we have 90% power to detect a minimal Cohen’s *d* effect size of 0.42 (alpha = 0.05, two-sided) in comparing 6-month changes in generalization and network flexibility between CDF and SFB. The Cohen’s *d* effect size of 0.42 is consistent with effect size estimates of 0.4–0.7 in the literature (Colcombe and Kramer, 2003). For Aim #2 Hypothesis 2.2, we performed power analysis using simulation studies, similar to Schoemann et al. (2017), to assure that our study has sufficient power to test simple mediation effects. Data were simulated assuming the effect sizes of the changes in generalization (Y) and the mediator (network flexibility) between CDF and SFB are both *d* = 0.42 (Aim1). 1,000 repetitions of simulations showed that our study with 122 subjects per group has >80% power to test a mediation effect of 0.13, assuming the correlation between Y and mediators >0.3, per prior studies. For Aim #3 Hypothesis 3, we have 90% power to test a Cohen’s *f*^2^ = 0.04 for comparing the 6-month changes in generalization and network flexibility between CDF and SFB in participants with ABCA7 rs3764650 non-risk vs. high-risk (alpha = 0.05, two-sided) [assuming the base rate for ABCA7 rs3764650 is 28.2%, per the Database of Single Nucleotide Polymorphisms (dbSNP) (Sayers et al., 2019) and 122 participants per group]. This effect size *f*^2^ = 0.04 is smaller than the Cohen’s *f*^2^ = 0.09 (Sinha et al., 2020) found in a prior study done by the PI in testing the interaction of time x treatment x genotype ABCA7. *Enrollment and Attrition Mitigation Plan*: Even if we have 10% fewer people (*n* = 110 subjects per group) complete the exercise program due to lower enrollment or higher attrition, we will still be able to show a main effect at *f*^2^ = 0.05.

Power for subgroup analysis. We expect the ratio for men to women to be about 1:2 (based on prevailing ratios in the community) and estimated the minimal effect size with alpha = 0.025 each for men (*n* = 40 per group) and women (*n* = 82 per group) before accounting for ~12.5% attrition. Aim #1 Hypothesis 1 and Aim #2 Hypothesis 2.1, we have 80% power to detect an effect size *d* of 0.7 in men and 0.49 in women for comparing 6-month changes in generalization and network flexibility between CDF and SFB. Aim #2 Hypothesis 2.2, similar to the power analysis for the full sample, we performed simulation studies assuming the effect sizes of the changes in generalization (Y) and the mediator (network flexibility) between CDF and SFB are both *d* = 0.8 in men and *d* = 0.53 in women. 1,000 repetitions of simulations showed that our study with 36 men per group has 80% power to test a mediation effect of 0.40 in men, assuming the correlation between Y and mediators >0.5, in women, we have 80% power to test a mediation effect of 0.21, assuming the correlation between Y and mediators >0.4. Aim #3 Hypothesis 3, With the high-risk variant of the ABCA7 genotype occurring in 28.2% of our participants, we will have 80% power to detect an effect size of *f*^2^ = 0.13 in men and *f^2^* = 0.06 in women.

### Study timeline

This investigation is funded by the National Institute on Aging. Enrollment will be completed on a rolling basis with participants joining both interventions every 6 weeks until September 2026. The expected enrollment completion date is August 2026. Data analysis will be completed from April–August 2027. The study outcomes and conclusions are expected to be completed in January 2028 and submitted for publication in February 2028.

## Discussion

To our knowledge, this is the first study examining the effect of a CDF intervention on generalization, MTL network flexibility, AD risk genetics, and blood-based biomarkers in older African Americans. Evidence suggests aerobic exercise interventions are effective at improving brain structure/volume, memory, and overall quality of life (Smith et al., 2010; Maass et al., 2015; Ogino et al., 2019). However, functional changes in the brain may precede structural changes (Bugg and Head, 2011; Hagan et al., 2016; Hsu et al., 2018; Reagh et al., 2018; Sinha et al., 2021). The current trial will help clarify whether structural changes in the hippocampus accompany functional changes in the MTL following participation in up to 69 60-min exercise sessions over a 24-week period. Further, exploring the effects of interventions on blood-based biomarkers of AD neuropathology will help us better understand whether exercise is AD modifying or merely compensating for pathological and non-pathological age-related cognitive decline. Furthermore, neuroprotective outcomes from enhanced cardiorespiratory fitness are not uniform due to, in part, genetics and social influences on health especially for African Americans who, on average, do not meet ACSM guidelines (150 min/week of moderate to vigorous physical activity) (He and Baker, 2005; Lines et al., 2014; Sinha et al., 2020). Moreover, personalized interventions may increase the robustness of the aerobic exercise effects in older African Americans. Cardio-dance group exercise classes are of interest because the social scaffolding and music creates a positive experience for participants. Thus, findings from this investigation will lay the foundation for future larger clinical trials to develop personalized exercise prescriptions for older African Americans that optimize the impact of this lifestyle intervention for improved brain health and AD and related dementias risk reduction.

### Limitations

This study will have limitations that impact interpretations. Our main focus is on improving fitness aerobic exercise and so we have not included other modes of exercise. The investigative team determined this was an acceptable limitation given the state of the field and the wealth of data using aerobic exercise as the primary mode. It should also be noted that the usage of a unconventional aerobic intervention (dance classes) requires a higher cognitive cost which will foster neural improvements independently of the aerobic-related adaptations. Additionally, using music and socialization in classes will have a positive impact on results and interpretations. Both classes will have music and socialization to ensure interpretations can only be made solely based on the mode of exercise administered. We also do not focus on other modifiable risk factors nor additional indirect benefits of aerobic exercise (e.g., reduced inflammation). Neither diet modifications, hypertension control, nor cognitive training are used in this study. This is relevant as large multi-domain interventions (e.g., exercise, diet and cognitive training) have reported improved brain health (Kivipelto et al., 2013; Ngandu et al., 2015; Vellas et al., 2015; van Charante et al., 2016; Rosenberg et al., 2018). Moreover, blood work is specific to the aims proposed and does not consider other vascular or endocrinological factors that could contribute to brain health among African Americans at this time. Moreover, we are focused on the medial temporal lobe (not other brain regions) because of its role in early stages of pre-symptomatic AD. Lastly, we are excluding ApoE *e4* allele from this study’s major aims. While this genotype has known profound negative effects on Alzheimer’s disease risk and negates neuroprotective benefits of aerobic exercise, we did not identify this within our preliminary data for this cohort of individuals. Nevertheless, a major strength of our study is that it is a double-blind randomized controlled trial featuring two exercise arms. This should strengthen the claim that CDF can enhance cognitive results better than the active comparator and negates socialization as a primary factor. Further, having a culturally tailored intervention decreases the likelihood of high dropout rates and promotes personalization of exercise. Lastly, using fMRI provide valuable insight toward brain adaptation compared with structural changes *per se*.

### Ethics and dissemination

This protocol was approved by the Internal Review Board of Rutgers, The State University of New Jersey (Pro2022001256). Informed consent will be obtained from participants upon arrival of study entry testing. Results and conclusions will be disseminated through presentations at academic conferences and publications in high impact peer-reviewed journals.

## Ethics statement

The studies involving humans were approved by Rutgers University Internal Review Board. The studies were conducted in accordance with the local legislation and institutional requirements. The participants provided their written informed consent to participate in this study.

## Author contributions

MG: Conceptualization, Funding acquisition, Investigation, Methodology, Supervision, Visualization, Writing – review & editing. JG: Investigation, Methodology, Project administration, Resources, Visualization, Writing – original draft, Writing – review & editing. BF: Conceptualization, Funding acquisition, Methodology, Resources, Visualization, Writing – original draft, Writing – review & editing. SM: Conceptualization, Funding acquisition, Methodology, Supervision, Writing – review & editing, Investigation. PD: Conceptualization, Funding acquisition, Methodology, Supervision, Visualization, Writing – review & editing, Investigation. KE: Conceptualization, Methodology, Visualization, Writing – review & editing. LH: Conceptualization, Funding acquisition, Methodology, Resources, Writing – review & editing, Investigation.

## Footnotes

^1^ www.worldcourier.com
